# Upregulated SMURF1 and miR-520d-5p in Basal Cell Carcinoma: Possible Implications for TGF-β/SMAD Signalling

**DOI:** 10.3390/ijms27219900

**Published:** 2026-11-05

**Authors:** Şükrü Kasap, Serkan Yaşar Çelik, Çilem Özdemir, Muhammed Said Aydın, Tuba Edgünlü

**Affiliations:** 1 Department of Plastic, Reconstructive and Aesthetic Surgery, Faculty of Medicine, Muğla Sıtkı Koçman University, 48000 Muğla, Turkey; 2 Department of Pathology, Faculty of Medicine, Muğla Sıtkı Koçman University, 48000 Muğla, Turkey; sycelik@gmail.com; 3 Department of Bioinformatics, Graduate School of Natural and Applied Sciences, Muğla Sıtkı Koçman University, 48000 Muğla, Turkey; 4 Department of Medical Biology, Faculty of Medicine, Muğla Sıtkı Koçman University, 48000 Muğla, Turkey

**Keywords:** basal cell carcinoma, *SMURF1*, miR-520d-5p, TGF-β signalling pathway

## Abstract

Basal cell carcinoma (BCC) is the most common type of skin cancer. SMAD-specific E3 ubiquitin protein ligase 1 (SMURF1), a protein involved in regulating the transforming growth factor-beta (TGF-β) signalling pathway, and miR-520d-5p may play a role in the development and progression of BCC. In this study, we investigated the potential roles of SMURF1 and miR-520d-5p, known to affect cancer pathogenesis, in the pathogenesis of BCC. We included 30 BCC tissues and 30 noncancerous skin tissues in the study. Gene expression levels were determined by quantitative polymerase chain reaction qPCR. We performed in silico analyses to identify possible genes and signalling pathways associated with BCC. SMURF1 and miR-520d-5p showed significantly increased expression in BCC tissues compared to controls (*p* = 0.029 and *p* = 0.011, respectively). According to in silico analyses, *SMURF1* and miR-520d-5p are predicted to be involved in the TGF-β signalling pathway. miR-520d-5p is predicted to regulate multiple target genes in the TGF-β signalling pathway, which requires experimental validation. Our findings indicate that *SMURF1* and miR-520d-5p are upregulated in BCC and may be associated with BCC pathogenesis. Both molecules are upregulated in BCC tissues and are implicated in the regulation of the TGF-β signalling pathway. According to in silico analysis, *SMURF1*, *SMAD2*, *SMAD4*, *TGFBR1*, *SMAD7*, *SMURF2*, *RUNX2*, *MAPK1*, and *TGFB3* are key genes involved in the TGF-β signalling pathway, which may be relevant to BCC pathogenesis.

## 1. Introduction

Basal cell carcinoma (BCC) is the most common malignant skin tumour. It exhibits local invasion and low metastasis [1]. There is ongoing debate about the cells that originate BCC [2]. Although BCC resembles the basal cell layer of the epidermis, it is thought to arise from cells in the hair follicle. This view is supported by the presence of certain proteins found in the hair follicle in BCC and by its association with the Sonic hedgehog gene. This hypothesis is also strengthened by the fact that BCC is rare in areas where there are no hair follicles. Patched Hedgehog gene mutations are associated with 30–75% of sporadic cases [3]. The cross-talk between the Hedgehog and TGF-β signalling pathways is evident, and genes in the TGF-β signalling pathway exhibit both tumour-suppressive and pro-oncogenic activity [4,5]. Although the Hedgehog/Patched pathway has been reported to be regulated by TGF-β family members, the cellular effects of altered protein levels are not well understood [6,7].

*SMURF1* is one of the Nedd4 family proteins expressed in many tissues, including nerve, lung, reproductive organs and bone. First, it was found to regulate bone formation by blocking the intracellular TGF-β/BMP (bone morphogenetic protein) pathway through ubiquitination and degradation of SMAD1 [8,9]. *SMURF1* plays an oncogenic role in tumour development, control of cell polarity, tumour cell invasion, and chemotherapy resistance [10]. Specifically, *SMURF1* expression in tumours was found to be significantly increased compared with matched normal tumour-adjacent tissue samples. Increased *SMURF1* expression was associated with vascular invasion, larger tumour size, distant metastasis, lymph node, advanced tumour-node-metastasis (TNM) stage, as well as decreased survival [11].

The miR-520 family has been reported to act as anti-onco-miRNAs by participating in the regulation of TGF-β and nuclear factor kappa B (NF-κB) signalling pathways in breast cancer [12]. The expression of miR-520d-5p was found to be decreased in cervical cancer tissues and cell lines. It has been shown that miR-520d-5p inhibits the activation of phosphoinositide 3-kinase (PI3K)/AKT signalling [13]. miR-520d-5p suppressed tumour growth and metastasis in colorectal cancer [14]. In addition, miR-520d-5p is predicted to target *SMURF1* (miRDB score 83/100), but this interaction has not been experimentally validated. Although previous studies reported changes in *SMURF1* and miR-520d-5p expression in different cancers, the roles of these genes for BCC are unclear. This study aimed to elucidate the potential effects of *SMURF1* and miR-520d-5p on BCC pathogenesis by molecular and in silico approaches.

## 2. Results

### 2.1. SMURF1 and miR-520d-5p Expression Levels

We determined SMURF1 and miR-520d-5p expression levels in skin tissues. SMURF1 and miR-520d-5p expression levels increased in BCC tissues compared to the control group. SMURF1 and miR-520d-5p expression levels were statistically significant between the BCC and control groups (*p* = 0.029 and *p* = 0.011, respectively) (Table 1 and Figure 1).

### 2.2. Correlation Analyses

To evaluate the relationship between target gene expressions and clinical/demographic parameters, Spearman rank correlation analysis was performed (Table 2). In the BCC group, no statistically significant correlation was observed between *SMURF1* and miR-520d-5p expression levels (ρ = 0.090, *p* = 0.644). Similarly, expression levels of both *SMURF1* and miR-520d-5p showed no significant correlation with patient age in either BCC or control groups (*p* > 0.05). Furthermore, neither *SMURF1* nor miR-520d-5p expression was significantly correlated with depth of invasion in BCC tissues (ρ = 0.075, *p* = 0.727 and ρ = 0.222, *p* = 0.298, respectively).

### 2.3. Subgroup and Multivariable Analyses of SMURF1 Expression According to Age and Sex

Subgroup analyses were performed to evaluate whether *SMURF1* expression varied according to age or sex within the BCC and control groups (Table 3). In BCC tissues, *SMURF1* expression did not significantly differ between participants aged ≤70 years and those aged >70 years (*p* = 0.580), and age was not correlated with *SMURF1* expression (ρ = −0.005, *p* = 0.981). In contrast, within the control group, participants aged >70 years exhibited significantly lower *SMURF1* levels compared to those aged ≤70 years (median 2.13 vs. 4.92; *p* = 0.047). However, this age-related difference was not observed in BCC tissues, and the group-by-age interaction in the linear model was not statistically significant (*p* = 0.675). Regarding sex-related differences, *SMURF1* expression showed no significant variation between females and males in BCC tissues (*p* = 0.214), controls (*p* = 0.531), or the overall cohort (*p* = 0.076). Furthermore, age-*SMURF1* correlations evaluated separately by sex were non-significant in both females (ρ = 0.033, *p* = 0.871) and males (ρ = −0.134, *p* = 0.481). In the multivariable linear regression model adjusting for age and sex, higher *SMURF1* expression in the BCC group remained statistically significant (*p* = 0.027), whereas neither age (*p* = 0.752) nor sex (*p* = 0.152) showed an independent significant association. Additionally, no significant group-by-sex (*p* = 0.573) or group-by-age (*p* = 0.675) interactions were detected. Given the exploratory nature of these subgroup analyses and the limited subgroup sample sizes, these comparisons were not adjusted for multiple testing.

### 2.4. In Silico Analysis

A series of in silico analyses confirmed our experimental hypothesis and findings. We used STRING (https://string-db.org/) for the potential interactions and functional enrichment analysis of SMURF1. The default minimum required interaction score was set to medium confidence (0.400). We determined the gene disease (C4721806) association with DisGeNET (https://www.disgenet.com/). We predicted the potential associations between miR-520d-5p and proteins in the STRING network using miRSystem (http://mirsystem.cgm.ntu.edu.tw/) and miRDB (http://www.mirdb.org/). As part of our study, we identified signalling pathways that could explain the association of hsa-miR-520d-5p with BCC using mirPath from the DIANA collection (https://dianalab.e-ce.uth.gr/).

We obtained STRING network and functional enrichment results for SMURF1-interacting proteins (Figure 2). The STRING network analysis revealed a complex interplay among SMURF1 and its interacting proteins, including TGF-beta receptor family members (TGFBR1, TGFBR2), the small GTPase RHOA, and SMAD proteins (SMAD1, SMAD2, SMAD5, SMAD7).

Functional enrichment analysis identified significant associations with biological processes related to cell signalling, particularly transmembrane receptor protein serine/threonine kinase signalling, growth factor signalling, and endogenous stimulus-response. Moreover, Reactome pathway analysis highlighted the involvement of *SMURF1* in the TGF-beta signalling pathway, including specific pathways such as “TGF-beta family member signalling” and “TGF-beta receptor signalling.” Additionally, enrichment in “TGF-beta signalling in EMT (epithelial to mesenchymal transition)” points to a potential role for SMURF1 in regulating EMT, a crucial process in cancer metastasis.

Molecular function enrichment revealed significant associations with SMAD binding, TGF-beta receptor binding, and activin binding, indicating that *SMURF1* and its interactors directly participate in these processes. Furthermore, the result related to E3 ubiquitin ligase binding supported that *SMURF1* may regulate TGF-beta signalling through ubiquitin-mediated protein degradation.

A total of 557 genes associated with BCC (C4721806) were identified in the DisGeNET database (Supplementary File S1). *SMAD2*, *SMAD7*, *TGFBR1*, *TGFBR2*, and *RUNX2* genes in the STRING network were also found to be included in the DisGeNET list. According to miRSystem findings, 782 target genes were predicted for hsa-miR-520d-5p. SMAD4, *SMURF2*, and *SMURF1* genes in the STRING network are miR-520d-5p targets. The TGFB3 gene, which is the DisGeNET list, is a target of miR-520d-5p according to the miRSystem.

We reported a total of 2404 target genes for hsa-miR-520d-5p according to miRDB. These genes include *SMURF2* (target score 98/100), *SMAD2* (target score 89/100), *RUNX2* (target score 61/100), *SMURF1* (target score 83/100), *TGFBR1* (target score 73/100), and *SMAD4* (target score 67/100) genes, located in the STRING network and the TGF-beta and SMAD signalling pathways. *MAPK1* (target score 100/100), *TGFB3* (target score 85/100), *TGFBI* (target score 74/100), and *TGFB2* (target score 72/100), which are among the BCC-associated genes in the DisGeNET database, are miR-520d-5p targets according to miRDB.

Additionally, mirPath searched the KEGG database using the microT-CDS approach and identified 28 pathways through which hsa-miR-520d-5p may affect genes. Among the prominent signalling pathways that could be associated with BCC, the TGF-beta signalling pathway ranked first. *FST*, *TGFBR1*, *SMAD2*, *PPP2CA*, *SMURF2*, *SMAD3*, *INHBA*, *ID4*, *ACVR1*, *ACVR2B*, *ZFYVE16*, *SMAD4*, *SMAD5*, *BMP2*, *SP1*, *ACVR1C*, *BMPR1A*, *MAPK1*, and *BMPR2* genes were targets of miR-520d-5p in the TGF-beta signalling pathway. Also, among the genes of these predicted target pathways, *FST*, *TGFBR1*, *SMAD2*, *SMAD4*, *BMP2*, *BMPR1A*, and *MAPK1* were identified as BCC-related genes by DisGeNET.

## 3. Discussion

Basal cell carcinoma (BCC) is the most common skin cancer, characterised by its local invasiveness and relatively low metastatic potential. The TGF-β pathway plays a critical role in regulating cellular processes such as differentiation, apoptosis, and proliferation [15]. TGF-β signalling regulates Hedgehog pathway activity, which has been shown to affect BCC pathogenesis. In this study, *SMURF1* and miR-520d-5p expression was higher in BCC than in non-cancerous controls, and in silico analyses suggest that both may be linked to the TGF-β signalling pathway; whether they contribute to BCC development or progression remains to be determined. Our findings revealed significantly increased expression levels of *SMURF1* and miR-520d-5p in BCC tissues compared to non-cancerous controls (*p* = 0.029 and *p* = 0.011, respectively). The upregulation of *SMURF1* is consistent with previous reports of its oncogenic role in other malignancies [9,10,16,17,18,19], whereas miR-520d-5p has mostly been described as tumour-suppressive in other cancers [13,14,20,21]. In silico analyses suggest that *SMURF1* and miR-520d-5p may regulate the TGF-β signalling pathway, a critical pathway affecting cellular processes such as differentiation, apoptosis, and proliferation [16].

*SMURF1* has been shown to play an oncogenic role in various cancers by modulating SMAD protein levels through ubiquitination and degradation. SMURF1 directly interacts with Phosphatidylinositol-4-phosphate 5-kinase type I gamma (PIPKIγ), which is highly expressed in lung cancer tissues, leading to its degradation [22]. Inhibition of *SMURF1* in breast cancer reduced ER alpha-positive cell proliferation [17]. High *SMURF1* expression has been associated with invasion and poor prognosis in glioma [10]. *SMURF1* knockdown in breast cancer cell line inhibited mitogen-activated protein kinase kinase 1 (*MEK1)* and epidermal growth factor (EGF)-induced invasion and migration [18]. SMURF1-mediated ubiquitination of Rho GTPase-activating protein 26 (ARHGAP26) in ovarian cancer promoted migration and invasion [19]. Our results align with the existing literature that positions *SMURF1* as a critical regulator of oncogenic processes in various cancers. The upregulation of *SMURF1* in BCC underscores its potential involvement in these pathways, although its utility as a biomarker or therapeutic target requires further validation. Our in silico analysis also confirmed that *SMURF1* interacts with various SMAD proteins, including *SMAD1*, *SMAD2*, *SMAD5*, and *SMAD7*, which are important components of the TGF-β signalling cascade. These findings suggest a potential link between *SMURF1* and TGF-β/SMAD signalling in BCC; however, the functional significance of this relationship remains to be established. Further investigation of these interactions may reveal novel therapeutic targets for BCC.

Although miR-520d-5p exhibited tumour-suppressive activity in cervical cancer [13], gastric cancer [20], colorectal cancer [14], and hepatoma cancer cells [21], we found higher miR-520d-5p levels in BCC than in controls; this is an association and does not by itself demonstrate oncogenic activity. While miR-520d-5p has been overwhelmingly characterised as a tumour suppressor in most malignancies, reports indicating its potential oncogenic or elevated role in cancers remain extremely limited, with low-grade glioma being a notable exception [23]. Thus, its upregulation in BCC may reflect a context-dependent role, but elevated expression alone cannot be interpreted as evidence of oncogenic potential. Notably, the 3p strand derived from the same precursor, miR-520d-3p, was downregulated in melanoma tissues and suppressed melanoma cell proliferation by targeting anti-silencing function 1B histone chaperone (ASF1B) [24], which shows that members of this miRNA family can have protective, tumour-suppressive effects in skin cancer. Because the two strands have different targets and the cancer types differ, these findings are not directly comparable to ours, but they argue against inferring an oncogenic function from expression levels alone. Many miRNAs act as tumour suppressors or as oncomiRs depending on tissue type, cellular environment and the repertoire of expressed target genes, and a single miRNA may repress both tumour-suppressive and oncogenic transcripts, so that its net effect reflects the balance between them [25]. Our data therefore do not allow us to conclude that miR-520d-5p is oncogenic in BCC. Establishing such a role would require functional validation (e.g., gain- or loss-of-function studies) and analysis of its relationship with clinicopathological features such as histological subtype, perineural or perivascular invasion, mitotic index, and ulceration, which we could not evaluate in this cohort (see limitations).

In silico analyses predict that miR-520d-5p targets a considerable number of genes related to the TGF-β pathway, including *SMAD2*, *SMAD4*, *SMURF1*, *SMURF2*, and *TGFBR1*. These molecules are central modulators of TGF-β/SMAD signalling, which can be tumour-suppressive or tumour-promoting, depending on the cellular context [4,5], and interacts with Hedgehog signalling, the main driver pathway of BCC [6,7]. These predictions are, however, hypothesis-generating: target prediction algorithms rely mainly on sequence complementarity and yield many false-positive targets [26], so the increased miR-520d-5p expression in BCC does not demonstrate regulation of these genes or an effect on tumorigenic processes in BCC.

Notably, in silico prediction indicates that miR-520d-5p may target *SMURF1*, and an inverse relationship would be expected under canonical regulation. However, we observed a parallel elevation of both molecules in BCC tissues, and their expression was not significantly correlated (ρ = 0.090, *p* = 0.644 in BCC). Weak or absent correlations between miRNAs and their predicted targets have been reported in cancer tissues [27], and miRNA effects on individual targets are generally modest and may be masked in complex tissues [28,29]. Several explanations are possible. First, miR-520d-5p might be induced as part of a compensatory negative feedback response to elevated *SMURF1*; miRNA-mediated feedback and feedforward loops are recurrent regulatory motifs [30,31], but we have no direct evidence for such a loop in BCC, and this remains a hypothesis. Second, miRNAs often act by inhibiting translation, and their effect may be more evident at the protein level than at the mRNA level [28,29]; we measured *SMURF1* mRNA but not SMURF1 protein. Second, *SMURF1* is regulated by other miRNAs (for example, miR-17 in mesenchymal stem cells [32]) and by transcriptional mechanisms (for example, androgen receptor signalling [33]), and in BCC these inputs may outweigh the effect of miR-520d-5p. Third, both molecules may be co-upregulated by a shared upstream signal, such as activation of the Hedgehog or TGF-β pathway [6,7]; this is a hypothesis that was not tested in the present study. Finally, bulk formalin-fixed paraffin-embedded (FFPE) tissue contains both tumour and stromal cells, and expression in different cellular compartments may mask a relationship between the two molecules. Protein-level analysis of SMURF1 and luciferase reporter assays are needed to clarify whether miR-520d-5p directly regulates *SMURF1* in BCC.

We examined whether expression varied with age and sex, because *SMURF1* has been linked to ageing-related pathways [32] and to sex steroid receptor signalling: it facilitates estrogen receptor α signalling in breast cancer cells [17], and androgens upregulate *SMURF1* transcription through the androgen receptor in prostate cancer cells [33]. In our cohort, higher *SMURF1* expression in BCC was independent of age and sex, with no significant group × age or group × sex interaction. Within BCC, *SMURF1* was not related to age or sex. In controls, *SMURF1* tended to be lower in participants older than 70 years, but this exploratory observation did not survive multiple-testing correction, depended on the age cut-off, and rested on ten older controls; it therefore requires confirmation. Females showed numerically higher *SMURF1* values in all strata, but this was not significant and was not independent of study group, and receptor expression and hormonal status were not assessed, so no conclusion about hormonal regulation can be drawn.

For miR-520d-5p, the difference between BCC and controls was larger at older ages (group × age interaction *p* = 0.022), reflecting an upward age trend in BCC and a downward trend in controls. Because neither trend was significant on its own, the interaction was not significant after correction for the four interaction tests, and the control group consisted of nevus-margin tissue rather than age-matched healthy skin, this finding is hypothesis-generating. If confirmed, it would suggest that the miR-520d-5p increase in BCC is more pronounced in older, cumulatively sun-exposed skin, which would be consistent with a role of ultraviolet exposure [34,35]. It does not demonstrate an oncogenic function.

This study has several limitations that should be acknowledged. First, the relatively small sample size (30 BCC and 30 non-cancerous skin tissues) may limit the statistical power and generalisability of our findings. Furthermore, all BCC lesions were obtained from the facial region, which may restrict the applicability of our results to tumors arising at other anatomical sites. Second, skin phototype and several clinicopathological characteristics, including histopathological subtype, perineural and perivascular invasion, mitotic index, ulceration, and history of solar-radiation exposure, were not systematically documented in the archived patient records and could not be retrieved retrospectively. Consequently, their potential associations with *SMURF1* and miR-520d-5p expression could not be evaluated, and only invasion depth was analysed. In addition, detailed quantitative comparisons among individual BCC subtypes were not feasible because of the limited sample size and the diversity of histological presentations. Although ultraviolet exposure is a major environmental risk factor for BCC [34,35], all lesions in this study originated from chronically sun-exposed facial skin, which may have reduced variability in anatomical site and exposure patterns. Third, our molecular analyses were limited to mRNA expression levels, without protein-level validation or functional assays. Although in silico analyses identified *SMURF1* as a potential target of miR-520d-5p, computational predictions alone cannot establish direct regulation, and potential pleiotropic or off-target effects of miR-520d-5p, which is predicted to regulate multiple transcripts within the TGF-β pathway, cannot be excluded. Future studies involving larger and more diverse cohorts, SMURF1 protein-level assessment, dual-luciferase reporter assays, overexpression and knockdown models, and rescue experiments using miRNA mimics or inhibitors in BCC cell lines are warranted to validate these findings and establish the direct regulatory relationship and biological relevance of the miR-520d-5p–*SMURF1* axis in BCC.

## 4. Material and Methods

### 4.1. Study Groups

Formalin-fixed paraffin-embedded (FFPE) tissues found in the archives of Muğla Sıtkı Koçman University Department of Pathology were used in the study. Cancerous tissues diagnosed with BCC (*n* = 30) were included as the patient group, and non-lesional surgical margin tissues diagnosed with melanocytic nevus (*n* = 30) were included as the control group. All of the tissues were taken from the facial area. The BCC group consisted of 16 females and 14 males, with a mean age of 72.42 ± 11.24 years, while the control group included 12 females and 18 males, with a mean age of 69.55 ± 12.66 years. Invasion depth was mean 0.27 ± 0.14 mm for the BCC group. There were no statistically significant differences in gender distribution or age between the two groups (*p* = 0.301 and *p* = 0.357, respectively).

The study protocol was approved by the Muğla Sıtkı Koçman University Faculty of Medicine Medical Ethics Committee (Approval number 10; Date: 8 January 2025). All procedures performed in studies involving human participants were in accordance with the ethical standards of the institutional and/or national research committee and with the 1964 Helsinki declaration and its later amendments or comparable ethical standards.

### 4.2. Deparaffinisation of Tissues

Three sections (each section 10 microns) were taken from the paraffin blocks. Xylene was used to remove paraffin from the tissue sections [36]. 1 mL of xylene was added to the tissues. The tubes were incubated at 60 °C for 5 min and centrifuged at 17,000× *g* for 2 min. In the final stage, the tissues were washed with 1 mL of 100% ethanol and phosphate-buffered saline (PBS), respectively.

### 4.3. SMURF1 Gene and miR-520d-5p Expression Analysis

RNA was isolated from FFPE tissues using TRIzol reagent (Invitrogen, Carlsbad, CA, USA). The High-Capacity A.B.T.TM cDNA Synthesis Kit (Cat. No: C03-01-20; Atlas Biyoteknoloji, Ankara, Türkiye) was employed to convert whole RNA into cDNA, following the instructions provided in the kit. The *SMURF1* and miR-520d-5p were amplified in accordance with the instructions provided in the Ampliqon RealQ Plus 2× Master Mix Green (Cat. No: A323402; Ampliqon A/S, Odense M, Denmark) reagent kit, using the Applied Biosystems StepOnePlus Real-Time PCR Detection System (Applied Biosystems, Foster City, CA, USA). *GAPDH* was employed as an endogenous control for *SMURF1*, while U6 was used for hsa-miR-520d-5p [37]. The 2^−ΔΔCT^ method was used to assess the relative gene expression, which came from a minimum of three separate studies [38]. The primer sequences and genes details are shown in Table 4.

### 4.4. Bioinformatic Analyses

No meta-analysis was performed; all in silico analyses were conducted using publicly available web-based tools. Protein–protein interaction (PPI) and functional enrichment analyses of SMURF1 were performed using STRING v12.0 (https://string-db.org/) for Homo sapiens in August 2026. All available interaction sources (text mining, experiments, databases, co-expression, neighbourhood, gene fusion, and co-occurrence) were included, with a minimum required interaction score of 0.400 (medium confidence). Functional enrichment was assessed for Gene Ontology (GO) Biological Process, GO Molecular Function, and Reactome pathways. *p*-values were adjusted for multiple testing within each category using the Benjamini–Hochberg procedure. Terms with an FDR ≤ 0.05, signal ≥ 0.01, strength ≥ 0.01, and at least two proteins represented in the network were retained. Redundant terms were grouped using a term similarity threshold of 0.8 [39]. Gene–disease associations for basal cell carcinoma (UMLS CUI: C4721806) were retrieved from DisGeNET v21.0 (https://www.disgenet.org/) in August 2026. All available associations were included without applying a minimum gene–disease association (GDA) score threshold; all retrieved associations had scores ≥ 0.01, yielding a total of 557 genes [40]. Potential targets of hsa-miR-520d-5p among the proteins identified in the STRING network were predicted using miRSystem (version 17.0) [41] and miRDB (version 6.0) [42]. The resulting predicted target lists were subsequently intersected with the proteins identified in the STRING network and the DisGeNET disease-associated gene list. Pathway enrichment analysis for hsa-miR-520d-5p was performed using DIANA-miRPath v3.0, with KEGG pathways as the reference database and DIANA-microT-CDS as the target prediction algorithm. Pathways with an FDR-adjusted *p*-value < 0.05 were considered statistically significant.

### 4.5. Statistical Analysis

Statistical analyses were performed in R (version 4.5.1; R Foundation for Statistical Computing, Vienna, Austria). All statistical analyses and data visualisation were performed in R (version 4.5.1; R Foundation for Statistical Computing, Vienna, Austria). Data were imported with the readxl package (v1.5.0.1) and summarised with dplyr (v1.2.0). Cliff’s delta was calculated with effsize (v0.8.1). All remaining tests (Shapiro–Wilk, Wilcoxon–Mann–Whitney, Welch’s *t*-test, Fisher’s exact test, Spearman’s correlation, and linear regression) were performed with base R (stats package). No external reference datasets were used and no meta-analysis was performed; all analyses were based on the qPCR expression data generated in the present study.

Normality was assessed with the Shapiro–Wilk test. Because qPCR expression values were right-skewed and not normally distributed, expression levels were compared between BCC and control groups with the Wilcoxon–Mann–Whitney test and are reported as median [interquartile range, IQR] together with mean ± standard deviation. As effect-size measures we report Cliff’s delta (95% bootstrap CI, 5000 resamples) and the ratio of geometric means (95% CI, from Welch’s *t*-test on log-transformed values). Age, which was normally distributed, was compared with Welch’s *t*-test; sex distribution was compared with Fisher’s exact test. Associations between continuous variables (SMURF1, miR-520d-5p, age and invasion depth) were evaluated with Spearman’s rank correlation coefficient (95% bootstrap CI). To examine whether expression differed according to age and sex, participants aged ≤70 years were compared with those aged >70 years (cut-off ≈ the median age of the cohort, 70 years), and females with males, in the BCC group, the control group and the overall cohort, using the Mann–Whitney U test. In addition, linear regression models with log-transformed expression as the dependent variable were fitted to estimate the association of study group (BCC vs. control) with expression after adjustment for age and sex; results are given as fold-changes with 95% CIs. Group × age (continuous, mean-centred) and group × sex interaction terms were tested in separate models, and residual normality was checked. A two-sided *p* < 0.05 was considered significant.

## 5. Conclusions

This study investigated the expression of *SMURF1* and miR-520d-5p in basal cell carcinoma (BCC). Both molecules were significantly higher in BCC tissues than in non-cancerous controls, and this increase was independent of age and sex. Although *SMURF1* is a predicted target of miR-520d-5p, their expression was not inversely correlated, and we did not test this interaction experimentally; the parallel increase therefore remains unexplained and requires functional validation. In silico analyses predict that both molecules are linked to the TGF-β signalling pathway. Our findings indicate an association of *SMURF1* and miR-520d-5p with BCC, and they do not establish a causal or therapeutic role. Protein-level analysis, reporter assays, and functional studies in BCC models are needed before these molecules can be considered as potential biomarkers or therapeutic targets.

## Figures and Tables

**Figure 1:**
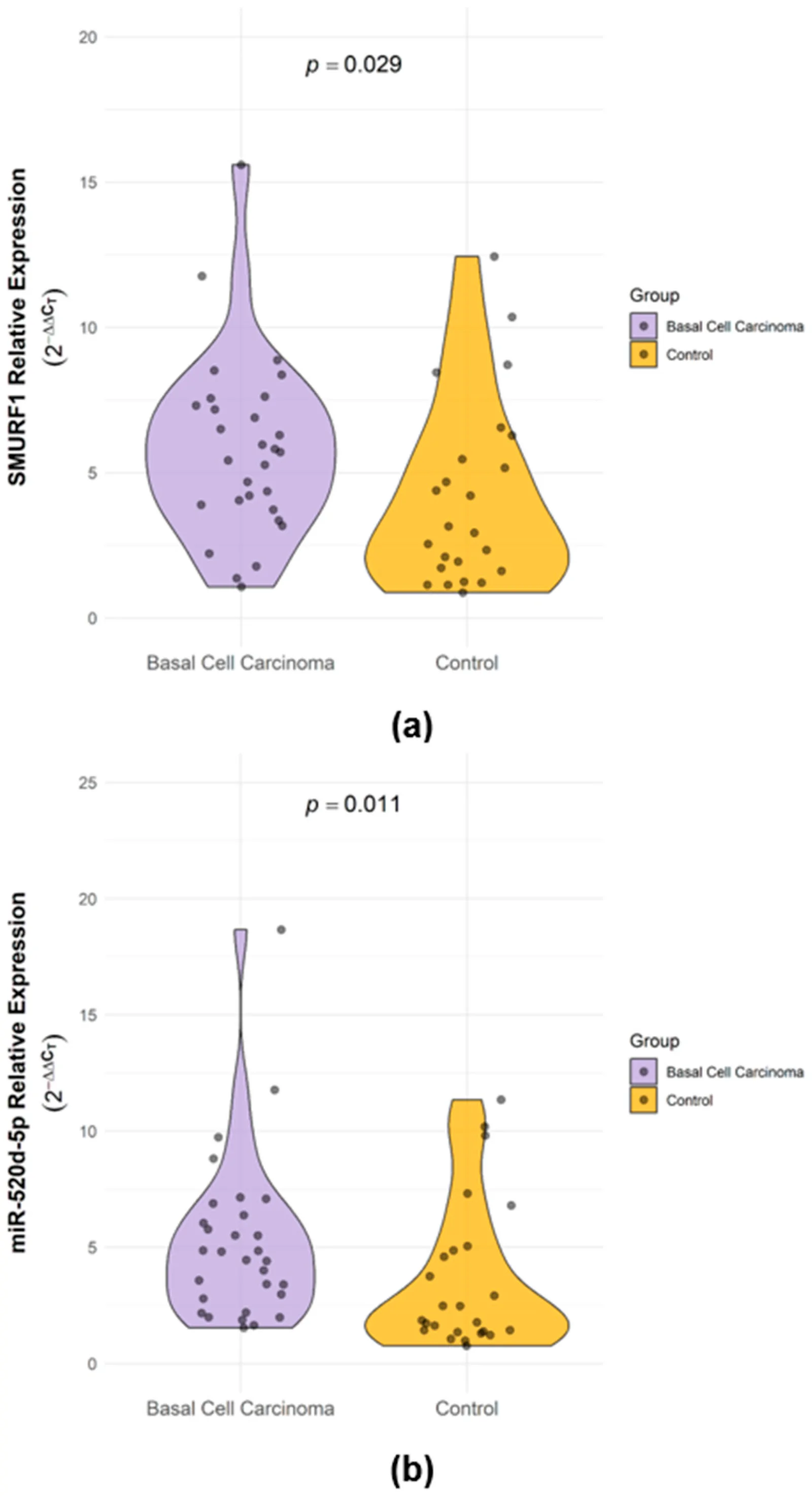
*SMURF1* (**a**) and miR-520d-5p (**b**) expression levels. The *SMURF1* and miR-520d-5p expression levels between BCC tissues and controls differed significantly (*p* = 0.029 and *p* = 0.011, respectively).

**Figure 2:**
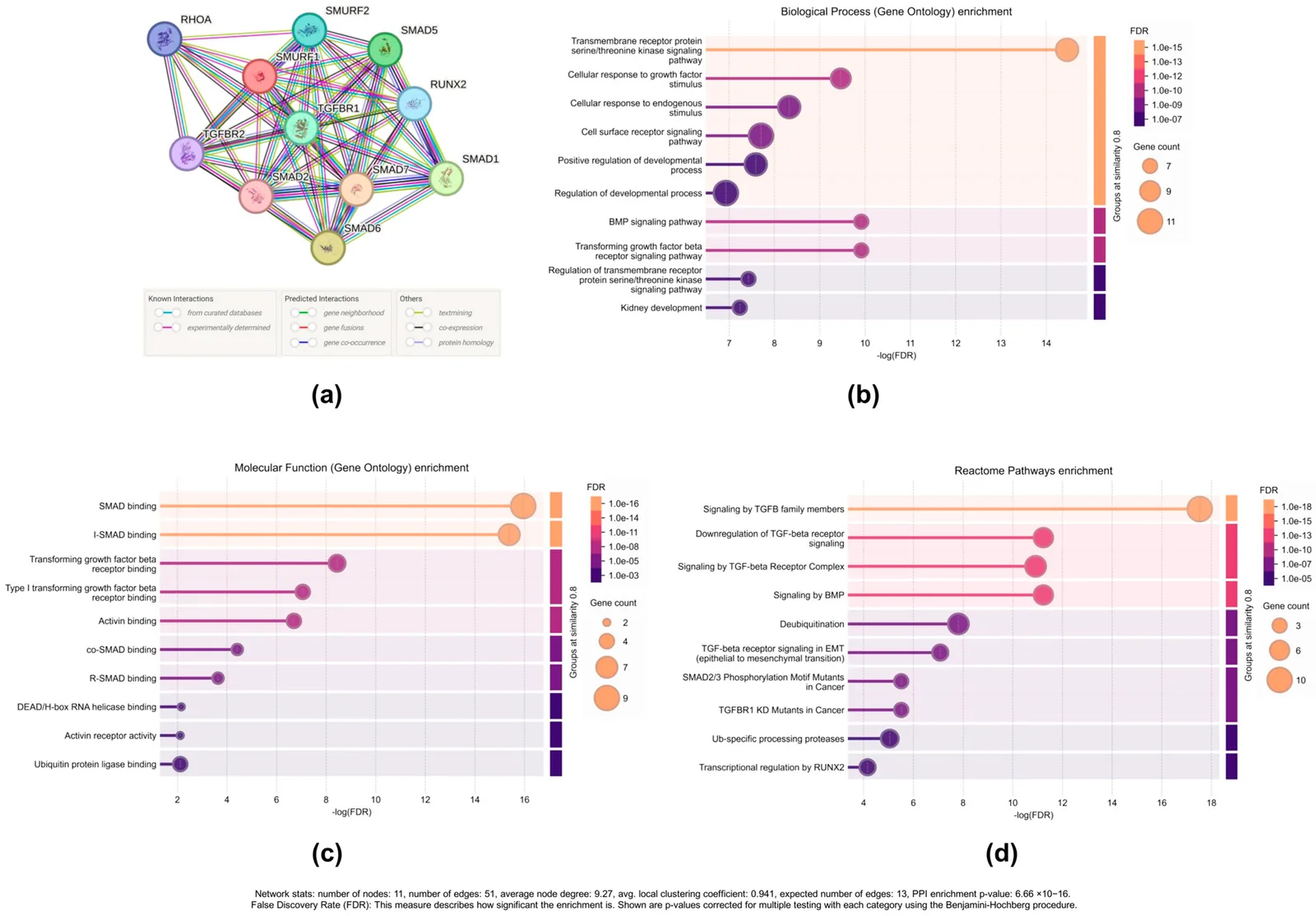
Functional enrichments of networks based on SMURF1 interactions: (**a**) interaction network, (**b**) GO biological process, (**c**) GO molecular function, (**d**) Reactome pathway database enrichment analysis results (Network stats: number of nodes: 11, number of edges: 51, average node degree: 9.27, avg. local clustering coefficient: 0.941, expected number of edges: 13, PPI enrichment *p*-value: 6.66 × 10^−16^; False Discovery Rate (FDR): This measure describes how significant the enrichment is. Shown are *p*-values corrected for multiple testing within each category using the Benjamini–Hochberg procedure).

**Table 1:** Expression levels of *SMURF1* and miR-520d-5p.

	Control (*n* = 30)		BCC (*n* = 30)		
Gene/miRNA	Mean ± SD	Median [25–75%]	Mean ± SD	Median [25–75%]	*p* *
*SMURF1*	4.21 ± 3.06	3.43 [1.64–6.07]	5.80 ± 3.10	5.71 [3.80–7.43]	0.029
miR-520d-5p	3.58 ± 3.16	1.86 [1.39–5.00]	5.21 ± 3.57	4.64 [2.49–6.64]	0.011

* Mann–Whitney test, SD: Standard deviation.

**Table 2:** Spearman rank correlation analysis between gene expression levels and clinical/demographic parameters in BCC and control groups.

Pair/Correlation Variables	BCC ρ	BCC *p*-Value	Control ρ	Control *p*-Value
*SMURF1*—miR-520d-5p	0.09	0.644	−0.157	0.465
*SMURF1*—Age	−0.005	0.981	−0.280	0.149
miR-520d-5p—Age	0.179	0.343	−0.206	0.322
*SMURF1*—Depth of Invasion	0.075	0.727	-	-
miR-520d-5p—Depth of Invasion	0.222	0.298	-	-

**Table 3:** Subgroup analysis of *SMURF1* expression by age and sex in BCC, control, and overall study groups.

Variable	BCC Group	Control Group	Overall Cohort
Age Group (years)			
≤70, median [IQR] (n)	6.16 [3.97–7.57]	4.92 [2.16–6.84]	5.32 [3.03–7.22]
>70, median [IQR] (n)	5.42 [3.89–7.17]	2.13 [1.33–3.94]	4.21 [1.99–5.83]
*p*-value (≤70 vs. >70) *	0.58	0.047	0.19
Age Correlation			
Spearman ρ (*p*-value)	−0.005 (0.981)	−0.280 (0.149)	−0.087 (0.520)
Sex			
Female, median [IQR] (n)	5.96 [4.89–7.10]	4.92 [1.60–7.03]	5.71 [4.13–7.10]
Male, median [IQR] (n)	4.28 [2.50–7.46]	3.04 [1.86–4.60]	3.72 [2.00–6.03]
*p*-value (Female vs. Male) *	0.214	0.531	0.076

IQR: Interquartile range; BCC: Basal cell carcinoma. * Comparisons performed using the Mann–Whitney U test.

**Table 4:** Primer sequences and amplicon sizes.

Gene/miRNA	Primers
SMURF1	F:5′-CACGGAATTTCACCATCAGC-3′ R: 5′-GTCCAGAAGCTGAAAGTCCT-3′
miR-520d-5p	F: 5′-TCTCAAACCGTAACCCACCA-3′ R: 5′-TGAGTCTACAAAGGGAAGCC-3′
GAPDH	F: 5′-GAAGGTGAAGGTCGGAGTC-3′ R: 5′-GAAGATGGTGATGGGATTTC-3′
U6	F: 5′-CTCGCTTCGGCAGCACA-3′ R: 5′-AACGCTTCACGAATTTGCGT-3′

## Data Availability

The data and materials in the current study are available from the corresponding author upon reasonable request.

## Supplementary Materials

The following supporting information can be downloaded at: https://www.mdpi.com/article/10.3390/ijms27219900/s1.
